# Oxidative stress and alopecia areata

**DOI:** 10.3389/fmed.2023.1181572

**Published:** 2023-06-15

**Authors:** Yi-qian Ma, Zhan Sun, Yu-Mei Li, Hui Xu

**Affiliations:** Department of Dermatology, Affiliated Hospital of Jiangsu University, Zhenjiang, China

**Keywords:** alopecia areata, oxidative stress, reactive oxygen species, antioxidants, autoimmune

## Abstract

Alopecia areata (AA) is an inflammatory autoimmune disease characterized by non-scarring hair loss on the scalp or any other part of the hair-bearing skin. While the collapse of the immune privilege is considered as one of the most accepted theories accounting for AA, the exact pathogenesis of this disease remains unclear by now. Other factors, such as genetic predisposition, allergies, microbiota, and psychological stress, also play an important role in the occurrence and development of AA. Oxidative stress (OS), an unbalance between the oxidation and antioxidant defense systems, is believed to be associated with AA and may trigger the collapse of hair follicle-immune privilege. In this review, we examine the evidence of oxidative stress in AA patients, as well as the relationship between the pathogenesis of AA and OS. In the future, antioxidants may play a new role as a supplementary therapy for AA.

## 1. Introduction

Alopecia areata (AA) is a non-scarring hair loss condition that affects people of all races, genders, and ages. The disease has a global prevalence of approximately 2%, with a prevalence of 0.27% in China (1).

While the exact pathogenesis of AA is not fully understood, it is believed to be a hair follicle-specific autoimmune disease caused by genetic and environmental factors.

During the anagen phase of hair growth, inflammatory cells such as CD8+ T cells, NK T cells, regulatory T cells, Th17 cells, and mast cells attack the hair follicles. This leads to the secretion of IFN-γ and an upregulation of NKG2D ligands in the hair follicle, which can expose hair follicle-associated autoantigens and trigger an autoimmune response in alopecia areata. This exposes anagen HF-associated autoantigens and HF-IP, resulting in the destruction of the hair follicle (2–4). Other factors such as genetic predisposition, allergies, psychological stress, and microbiota also play a role in the occurrence and development of AA (2).

Oxidative stress (OS) is characterized by an imbalance between oxidation and anti-oxidation mechanisms (enzymic and non-enzymic antioxidants), leading to an increase in oxidation intermediates such as reactive oxygen species (ROS) (5). The generation of abnormal ROS (lipid, protein, DNA free radicals etc.) has been associated with a variety of skin diseases, including AA. Therefore, in this review, we aim to discuss the evidence of oxidative stress in AA patients, as well as the relationship between oxidative stress and the pathogenesis of AA.

## 2. Evidence of oxidative stress in AA patients

### 2.1. Protein oxides in alopecia areata patients

Reactive oxygen species can lead to free radical reactions and the production of oxidized modified proteins. Bar-Or et al. (6) found that during myocardial ischemia, human serum albumin passing through the ischemic site experienced amino-terminal modification due to free radical damage, resulting in modified albumin, known as IMA. Ataş et al. (7) found that the mean serum level of IMA was higher in patients with AA than in the control group, suggesting IMA as a potential biomarker for oxidative stress in AA. Similarly, a case–control study conducted by Incel-Uysal et al. (8) reported significantly higher mean and adjusted IMA levels in AA patients than in the control group, indicating a potential association between IMA and the imbalance of oxidative stress. A newly released study conducted by Tanacan et al. (9) observed significant differences between the mean IMA and IMA/albumin ratio in AA patients. However, there is no clear evidence that IMA is related to the severity and duration of AA (9).

### 2.2. Lipid peroxides in alopecia areata patients

Malondialdehyde is the end-product of peroxidation reactions caused by ROS acting on lipids. Previous studies have observed the generation of lipid radicals in the skin of mice treated with PPIX plus natural light (10). MDA may be a major product of lipid oxidation and thus an important marker (11).

Several studies have measured MDA levels in the plasma and scalp biopsies of AA patients. Akar et al. (12) found significantly higher levels of TBARS (a marker for MDA) in scalp biopsy tissues of AA patients than in healthy subjects, with the average levels being two times higher in the early phase of the disease than in the late phase. Similarly, Cwynar et al. (13) found significantly higher plasma MDA levels in AA patients than in the control group. A meta-analysis conducted by Acharya et al. (14) also reported significantly higher plasma MDA levels in AA patients than in control subjects. Sachdeva et al. (15) found that the levels of MDA were positively correlated with the severity of AA.

### 2.3. Nucleic acid DNA damage products of alopecia areata patients

8-hydroxy-20-deoxyguanosine (8-OHdG) is the most commonly used biomarker of oxidative DNA damage. Mustafa et al. (16) reported significantly elevated serum 8-OHdG levels in AA patients compared with the control group and suggested that 8-OHdG could be an independent predictor of AA severity.

### 2.4. Enzymatic antioxidants in alopecia areata patients

Superoxide dismutase (SOD) is an enzyme that eliminates O_2_^•−^ and transforms it into H_2_O_2_ in the body. However, the results of studies on SOD activity in AA patients are contradictory. Akar et al. (12) found significantly higher levels of SOD activity in scalp biopsy tissues of AA patients than in the control group, with the mean level of SOD activity in the early phase being two times higher than in the late phase. In contrast, Yenin et al. (17) found significantly decreased SOD activity levels in erythrocytes of AA patients compared with the control group. Acharya et al. also reported lower serum SOD levels in AA patients than in the control group, while Sachdeva et al. found significantly decreased whole blood SOD levels in AA patients, with the levels decreasing as the severity of the disease increased (14, 15). These findings may be explained by the homing of inflammatory cells to AA lesions.

Glutathione peroxidase (GSH-Px) is an important H_2_O_2_ decomposition enzyme widely present in the human body. Yenin et al. (17) found that the activity of GSH-Px in the red blood cells of patients with alopecia areata was significantly lower than that of the control group.

Manganese superoxide dismutase (MnSOD) is an enzyme belonging to SOD, which is located in the mitochondrial matrix and can remove O_2_^•−^ generated by energy metabolism, while glutathione peroxidase 1 (GPX1) is the most abundant of the four identified GPX isozymes. The final level of mitochondrial ROS may depend on the activity of MnSOD and GPX1. Genetic differences in antioxidant genes encoding MnSOD and GPX1 may alter the detoxification effect of ROS and increase disease risk (18). Kalkan et al. (19) studied the possible association between MnSOD and GPX1 gene polymorphisms and susceptibility and disease progression of alopecia areata in a Turkish population. The results did not show a statistically significant correlation between clinical and demographic characteristics of alopecia areata patients and MnSOD Ala9Val and GPX1 Pro198 Leu polymorphic genes, but there was a significant difference between genders (19). The limitations of this study mainly include that most patients were composed of AA patients with less than 50% involvement of the scalp.

Ramadan et al. (20) reported that both tissue and serum levels of PON1 were significantly lower in patients with AA compared to controls, indicating a reduced capacity for antioxidant defense in these patients. Similarly, Dizen-Namdar et al. (21) found that serum levels of PON1 activity were significantly decreased in patients with AA compared to control groups. They also observed significantly higher levels of TOS and lower levels of TAC in patients with AA, indicating an imbalance between oxidative stress and antioxidant capacity.

### 2.5. Non-enzymatic antioxidants in alopecia areata patients

Vitamin E (tocopherol) is a major physiological barrier antioxidant (22). A tocopherol molecule can scour two free peroxides and prevent further oxidation of polyunsaturated fatty acids (PUFA) in cell membranes. It can also inhibit cyclooxygenase-2 (COX2) activity by reducing NO production, thereby reducing prostaglandin E2 (PGE2, an important pro-inflammatory mediator). So vitamin E is the most important non-enzymatic antioxidant in the skin (23). Ramadan et al. (20) found that the tissue and serum levels of vitamin E in patients with alopecia areata were significantly lower than those in the control group.

Vitamin D regulates the growth and differentiation of hair follicle keratinocytes by binding to the nuclear vitamin D receptor (VDR). It also has anti-inflammatory and immunomodulatory effects (24, 25). 1,25(OH)2D inhibits T cell differentiation to Th1 type, reduces the secretion of inflammatory cytokines (IL-2, IFN-γ, TNF-α), promotes Th2 differentiation and increases the secretion of inflammatory cytokines (IL-4, IL-5, and IL-10) (26). These effects may be related to autoimmunity of alopecia areata. Recent experiments have shown that vitamin D is also an antioxidant (27). Compared with placebo, vitamin D supplementation significantly increased TAC and GSH levels in patients on methadone maintenance therapy (an alternative treatment for people addicted to opioid abuse) (28). Sachdeva et al. (15) found that serum vitamin D levels were significantly lower in patients with alopecia areata compared with controls; they also noted that low vitamin D levels might be an important risk factor for increased disease severity in patients with alopecia areata. A systematic review and meta-analysis by Lee et al. (29) also demonstrated low serum 25-hydroxyvitamin D levels in patients with alopecia areata, but did not find a clear correlation between serum 25-hydroxyvitamin D levels and the degree of hair loss.

In addition to playing a role in enzymatic antioxidant systems, certain metals also contribute to non-enzymatic antioxidant systems. For example, zinc is a crucial component of the SOD enzyme system, while lead is involved in the electron transport chain of ROS. In a case–control study by Ozaydin-Yavuz et al. (30), it was found that patients with alopecia areata had significantly lower levels of serum zinc and manganese, while their serum lead, iron, magnesium, cobalt, and copper contents were 10 times higher than those of the control group. This suggests that patients with alopecia areata may have a stronger reactive oxygen production response, leading to the depletion of metal elements required for enzymatic antioxidants in the body.

## 3. Relationship between the pathogenesis of alopecia areata and oxidative stress

### 3.1. Oxidative stress and alopecia areata

The skin is one of the main barriers between the environment and the body. It is constantly exposed to a variety of physical and chemical influences and is therefore most directly affected by ROS, which are produced when skin is exposed to visible light or ultraviolet rays (10). Then as a result of the free radical chain reaction, the skin produced lipid, protein, DNA, and other free radicals. The direct effects of ROS and these free radicals on skin are unclear. However, oxidants produced by ROS, including 4-HNE and MDA, change the structure of proteins, thereby inducing apoptosis and regulating the release of inflammatory cytokines (5). In addition, ROS could also damage the skin in the following ways: activating transcription factors, upregulating the expression levels of EGF receptor in human keratinocytes (31), inducing ferroptosis etc. (32). ROS can trigger and maintain the autoimmune cascade by enhancing cell apoptosis and reducing the efficiency of its clearance, and the resulting accumulation of apoptotic residues promotes the formation of auto-antibodies (33). Elevated levels of endogenous oxidative stress may also be related to disease burden, medication regimen, and duration of treatment, age, and excessive physical and emotional stress associated with comorbid medical conditions (34).

Fortunately, there is an antioxidant system in our body to help skin eliminate ROS and free radicals. The antioxidant system consists of enzymic and non-enzymic antioxidants, which complement and depend on each other to maintain the balance between oxidation and antioxidant. SOD, CAT, and GSH-Px are typical enzymatic antioxidants. Non-enzymatic antioxidants include vitamin E, vitamin C, vitamin D, GSH, β-carotene, etc. Oxidative stress may be an important trigger for the development of various diseases, such as systemic lupus erythematosus (SLE), rheumatoid arthritis (RA), and Type 1 diabetes mellitus (DM 1). Resulting from the constant attack of ROS on the skin, oxidative stress has been increasingly investigated as a potential contributor to the development of autoimmune skin diseases, including alopecia areata. The role of oxidative stress in alopecia areata has been increasingly investigated in the recent literature. As such, investigating the role of oxidative stress in AA is an important area of research that could have implications for treatment and prevention.

### 3.2. Oxidative stress may trigger the collapse of hair follicle-immune privilege

Hair follicles are immune privilege organs, which prevent their own antigens from being presented to CD8 + T cells by downregulating the expression of MHC class I molecules in anagen hair follicles. Collapse of HF-IP is one of the most widely accepted theories about the pathogenesis of alopecia areata. Collapse of HF-IP may be characterized by upregulation of MHC-I and MHC-II expression (3). NK cell receptor D (NKG2D) was mainly expressed on the surface of NK cells, NK T cells, γσT cells, and CD8 + T cells and the gene encoding NKG2D is upregulated in patients with AA (35). NKG2D can recognize MICA/B (soluble MHC Class I molecule-related protein), which is the only receptor that can be recognized with MICA/B found *in vivo* so far. As an activator receptor, NKG2D mediates the activation of above immune cells and then kills target cells. IFN-γ and CD8+ NKG2D+ T cells play a critical role in the collapse of IP (34). Hsp60, Hsp70, and gp96 can enhance the expression of B7 and MHC-II on the surface of plasmacytoid dendritic cells (antigen presenting cells), stimulate dendritic cell maturation, migration to draining lymph nodes, and induce the secretion of chemokines by macrophages and plasmacytoid dendritic cells (DCs) (36). Studies have shown that IFN-γ secreted by cytotoxic T lymphocytes around vitiligo lesions can up-regulate the expression of Hsp70, and Hsp70 can also enhance the release of IFN-γ by cytotoxic T lymphocytes through a positive feedback mechanism (37). There is a surprising similarity between the pathogenesis of vitiligo and alopecia areata, and the above process may also occurs around the lesions of alopecia areata.

**Figure 1:**
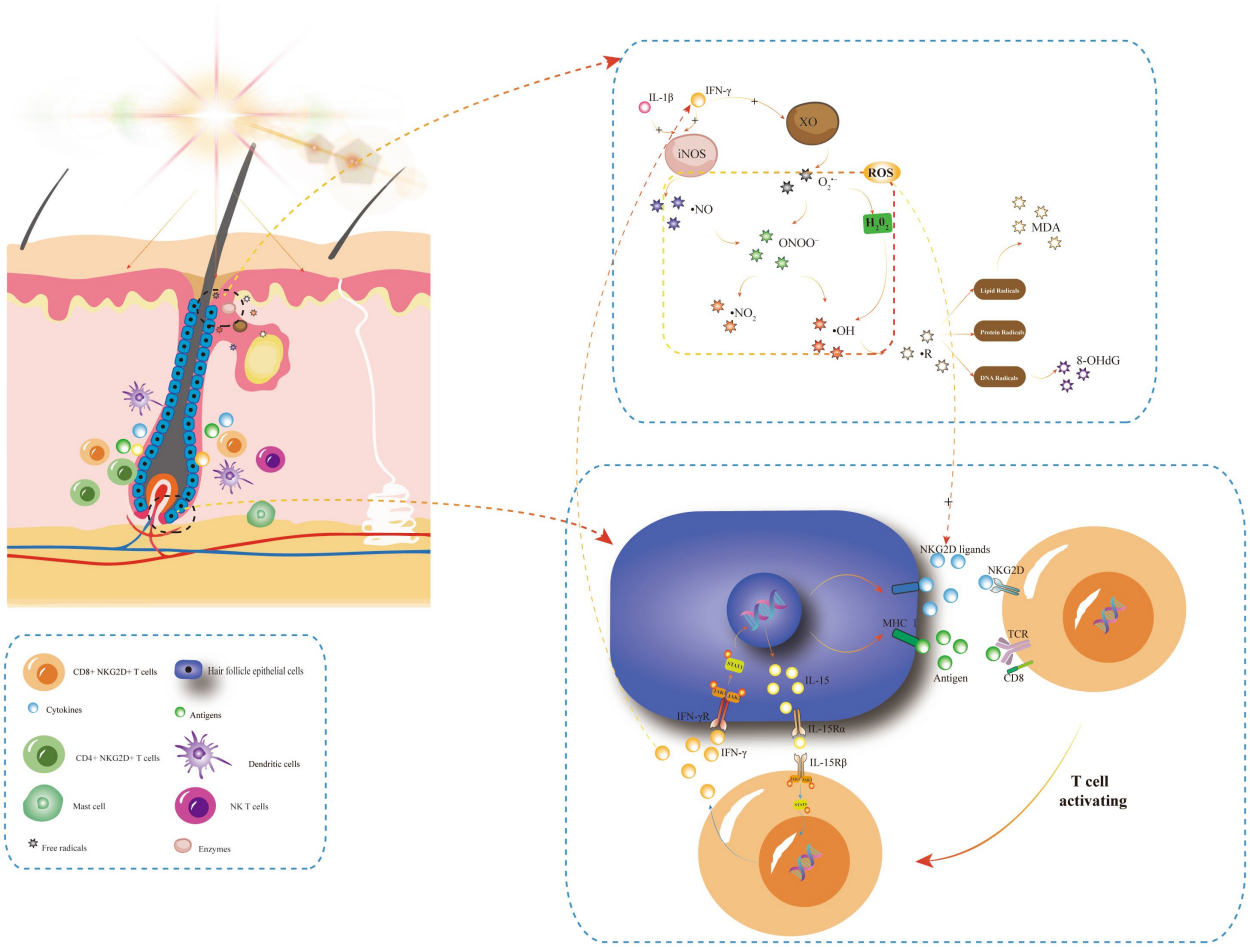
The positive feedback-loop of losing hair follicle-immune privileges and its relationship with oxidative stress. In AA, stimulating factors such as UVB or natural light irradiation can induce the chemotaxis of various inflammatory cells around hair follicular epithelial cells (HFECs). HFECs upregulate the expression of MHC and NKG2D ligands, and then NKG2D + CD4 + T cells become effector T cells after binding to MHC I antigen complex and NKG2D ligands on its surface. Activated T cells produce IFN-γ via JAK1 and JAK3, and IFN-γ stimulates IL-15 production in HF-ecs via JAK1 and JAK2. Then, IL-15 again binds to CD8 + NKG2D + T cells to produce more IFN-γ, forming a positive feedback loop that leads to the collapse of immune privilege of hair follicles and promotes the autoimmune attack of hair follicles by inflammatory cells, such as dendritic cells, CD4 + T cells, NK T cells, and mast cells. IFN-γ and IL-1β are oxidative stress triggers, and IFN-γ is a key player in XO-mediated oxidative stress. Light irradiation can induce an increase in XO enzyme activity, producing more oxygen free radicals. In addition, skin inflammation caused by UV irradiation synthesized large amounts of ·NO in the presence of the iNOS enzyme. IFN-γ and IL-1β can induce the rapid expression of iNOS and lead to O_2_^•−^ produced by XO forming ONOO−, which then decompose into ·NO2 and ·OH, causing free radical reactions, including lipid free radicals (MDA, etc.), protein free radicals and DNA free radicals (8-OHdG, etc.). These ROS can promote the upregulation of NKG2D ligands such as ULBP, which triggers the loss of immune immunity of hair follicles and promotes the development of AA. INFγ, interferon gamma; STAT, signal transducer and activator; P, phosphorylated; TCR, T cell receptor; NKG2D, NK cell receptor D; XO, xanthine oxidase; and iNOS, inducible nitric oxide synthase.

Recent studies have shown that intravenous injection of IFN-γ could lead to alopecia areata in young C3H/HeJ mice. In contrast, IFN-γ -deficient mice were less susceptible to alopecia areata (2). CD8 + T cells (a type of cytotoxic T lymphocyte) expressing NKG2D bind to NKG2D ligands produced by HFECs to form MHC-I antigen complexes and thus become effector cells. CD8+ NKG2D+ T cells produce IFN-γ through JAK1 and JAK3 pathways, which stimulates HFECs to produce IL-15. Then this interleukin binds CD8+ NKG2D+ T cells to further stimulate IFN-γ production through JAK1 and JAK3 pathways, forming a positive feedback loop (Figure 1) that destroys anagen hair follicles and leads to alopecia areata (34).

Besides CD8+ NKG2D+ T cells and IFN-γ, some inflammatory cells and cytokines also play a significant role in the immune pathogenesis of alopecia areata, such as dendritic cells, CD4 + T cells, NK T cells, mast cells and eosinophils, and other cytokines such as IL-2, CXCLs, and TNF-α. IL-15 also stimulates the proliferation and activation of T cells, macrophages, and CD5 memory lymphocytes. They interact with each other to promote the progress of hair follicle inflammation.

IFN-γ, IL-1β, and IL-6 are known as oxidative stress triggers. It has been shown that IFN-γ is a key player in xanthine oxidase (XO)-mediated oxidative stress. Light irradiation can induce an increase in XO activity in epidermal keratinocytes and endothelial cells and produce more oxygen free radicals (38). In addition, XO and its oxidative stress products are also involved in the innate immunity of the skin. XO appears in the early stage of skin inflammation caused by lipopolysaccharides (LPS, a strong stimulator of toll-like receptors) (39).

The activity of inducible nitric oxide synthase (iNOS) was also significantly increased in LPS-induced inflammatory skin disease mouse models. iNOS are mainly found in fibroblasts, and a large amount of ·NO is synthesized in skin healing and skin inflammation caused by UV irradiation (40, 41). In the presence of a large amount of ·NO, O_2_^•−^ produced by XO generates ONOO−, which then decomposes into nitrogen dioxide free radicals (NO_2_) and OH, which in turn nitrates tyrosine residues and affects intracellular signaling (42). IFN-γ and IL-1β can induce the rapid expression of iNOS (Figure 1) (43). Studies have shown that oxidative stress upregulates the expression of intercellular adhesion molecule 1(ICAM-1) in epithelial cells through an IL-6/AKT/ST4T3/NF-KB dependent pathway, and its upregulation is closely related to pro-inflammatory cytokines such as IL-6, IL-1, and TNF-α (44). Tomaszewska et al. (45) found that serum levels of IFN-γ, IL-1β, and L-6 were significantly increased in patients with AA, and the elevated levels of IL-6 serum were proportional to the duration of AA. However, there was no correlation between the levels of these three serum cytokines and disease degree (SALT) and disease activity (VIDA) (45).

Oxidative stress has been shown to upregulate NKG2D ligands, including MICA and ULBP, which may trigger the collapse of hair follicle immune privilege (HF-IP) and contribute to the development of alopecia areata (4). Interestingly, studies have found that ULBP6 and ULBP3 are NKG2D ligands specifically associated with alopecia areata. More importantly, SNPs of antioxidant coding genes PRDX5 and ALDH2 have been identified as associated with the alopecia areata phenotype. Some studies hypothesized that these two SNPs hypotheses may interfere with the hair growth cycle in affected individuals (46, 47).

### 3.3. Autophagy and oxidative stress in AA patients

Recently, many studies have focused on the role of autophagy in AA. Interfering with autophagy in AA may impair the clearance of dead cell debris; disrupt active hair growth in anagen, which leads to skin tissue inflammation (48). Petukhova et al. (49) found that the expression level of ATG4B, a gene involved in autophagy, is altered in AA patients. Simultaneously, autophagy plays a crucial role in antigen processing or presentation, which could reduce the expression of MHC class I molecules, which are abnormally downregulated in AA (50). Gund et al. (51) demonstrated that autophagy was blocked in C3H/HeJ AA mice, and induction of autophagy delayed the development of alopecia, what is more, inhibition of autophagy accelerated the development of alopecia. Hardman et al. (52) also showed that autophagy activity was blocked in traumatic HF in AA patients. Autophagy protects cells from ROS by degrading and recycling damaged cellular components. In response to oxidative stress, ubiquitination of ATG9A (a multi-spanning transmembrane protein) induces autophagy (53).

### 3.4. Psychological stress and oxidative stress in AA patients

Psychological stress, such as depression and anxiety, has been reported to be more common in patients with AA than in healthy individuals. Their psychological state can be assessed by clinical psychiatric evaluation such as hospital anxiety scale score (HADS-A score), and depression scale score (HADS-D score), among others. This is attributed to the shame and impaired self-esteem caused by hair loss, which can affect appearance (54, 55). Although it is still controversial whether depression and anxiety are pathogenic factors of AA, some studies suggest that patients who experienced acute psychological stress events for a long time (mostly in childhood), such as academic pressure or death of close family members, may have a higher risk of developing AA. The pathophysiological mechanism of hair loss induced by psychological stress may be associated with the upregulation of corticotropin-releasing hormone receptor (CRHR), adrenocorticotropin (ACTH), and acetylcholine (ACh) in the skin, which regulate immunity, promote cytokine secretion, and lead to the collapse of HF-IP (56–58).

Since both oxidative stress and psychological stress can trigger the collapse of HF-IP to cause alopecia areata, the relationship between these two factors needs to be explored. Can depression, anxiety, or other mental states lead to further increase in oxidative stress levels in AA patients? A recent case–control study by Cakirca et al. (59) investigated the effect of anxiety and depression symptoms on oxidative stress in AA patients. The study found that although total oxidation state (TOS), total antioxidant capacity (TAC), depression, and anxiety scores were significantly increased in AA patients compared to the control group, there was no significant difference between the scores and the TAS or the TOS levels of patients (59). Hence, larger sample sizes, more extensive research, and more scientific evaluation criteria are needed to explore the interaction between psychological stress and oxidative stress.

## 4. The application of antioxidant in the treatment of alopecia areata

The above studies not only indicate that oxidative stress may be one of the pathogenesis of alopecia areata, but also suggest that antioxidants can be used as an adjuvant therapy for alopecia areata, especially for patients with mild to moderate alopecia areata. Currently, the treatments for alopecia areata mainly include glucocorticoids, contact immunotherapy, minoxidil, immunosuppressants, etc. (2). Continued use of glucocorticoids may increase the chances of patients experiencing side effects, such as infections, ulcers, osteoporosis, and elevated blood sugar. While the benefits of single antioxidant therapy may be limited and may even have negative effects, for example, long-term use of beta-carotene may increase the incidence of lung cancer in smokers (60), while vitamin E may increase the risk of bleeding in people with oral anticoagulants and even reduce the lipid-lowering effect of statins (61), Therefore, the safety and efficacy of long-term additional oral antioxidants deserve further exploration. But in the case of alopecia areata, antioxidants as a form of combination therapy with steroids may help reduce steroid use. Calcipotriol is a vitamin D derivative. Narang et al. (62) found that patients with alopecia areata were treated with 0.005% calcitriol lotion for 3 months with significant improvement in disease severity. Alam et al. (63) also found that external use of 0.005% calcipotriol ointment combined with external use of 0.1% mometasone cream had better efficacy in the treatment of alopecia areata than external use of mometasone alone. However, studies such as double-blind clinical trials with larger samples are needed to further demonstrate the efficacy of vitamin D analogs in combination with other traditional therapies in patients with alopecia areata. In 2020, Abbas conducted a case–control study where he found that oral ginger powder, a powerful antioxidant, was effective in treating alopecia areata, significantly improving the antioxidant/oxidative balance of red blood cells and lymphocytes in patients with alopecia areata compared to healthy subjects. In addition, ginger powder can also increase the serum zinc concentration, even making it reach the serum zinc level of healthy control group (64).

## 5. Conclusion

Alopecia areata is an immune disease caused by both genetic and environmental factors, and its pathogenesis has not been fully clarified. Oxidative stress may be one of the initial factor triggering immune disorders in patients with AA, and eventually lead to varying degrees of hair loss. Many studies have demonstrated a close link between AA and oxidative stress. However, the relationship between alopecia areata and oxidative stress is quite complex, and more solid and direct evidence is needed. The common disadvantage of these studies is that they only include a small number of cases. More importantly, more real-world studies are needed to demonstrate the possibility and reliability of antioxidants as adjuvant therapies for alopecia areata. Based on previous findings, antioxidants may be a promising adjuvant therapy when combined with other traditional treatments for AA.

## Author contributions

Y-qM: conceptualization, methodology, software, investigation, and writing—original draft. ZS: data curation, investigation, and writing—original draft. Y-ML: supervision, validation, and writing—review and editing. HX: conceptualization, methodology, supervision, and writing—review and editing. All authors contributed to the article and approved the submitted version.

## Funding

This research was funded by “the National Key Research and Development Programs of China, grant number 2022YFC2603801,” “the Maternal and Child Health Project of Jiangsu Province (F201717),” “the Doctor Project of Affiliated Hospital of Jiangsu University (jdfyrc2019003),” and “Clinical and Virology Study of 2019-ncov Infection in Patients with Moderate to Severe Psoriasis (Jdfyxgzx005).”

## Conflict of interest

The authors declare that the research was conducted in the absence of any commercial or financial relationships that could be construed as a potential conflict of interest.

## Publisher’s note

All claims expressed in this article are solely those of the authors and do not necessarily represent those of their affiliated organizations, or those of the publisher, the editors and the reviewers. Any product that may be evaluated in this article, or claim that may be made by its manufacturer, is not guaranteed or endorsed by the publisher.

## Abbreviations

AA: Alopecia areata
HFECs: Hair follicular epithelial cells
IP: Immune privilege
HF: Hair follicle
ROS: Reactive oxygen species
MHC: Major histocompatibility complex
4-HNE: 4-Hydroxy-2-nonenal
MDA: Malondialdehyde
GSH: Glutathione
TBARS: Thiobarbituric acid-reactive substances
CAT: Catalase
GSH-Px: Glutathione peroxidase
SNPs: Single nucleotide polymorphisms
IMA: Ischemia-modified albumin
8-OHdG: 8-Hydroxy-20-deoxyguanosine
SOD: Superoxide dismutase
PON1: Paraoxonase 1
TOS: Total oxidative status
TAC: Total antioxidant capacity
